# Extended use of the wearable cardioverter-defibrillator in patients at risk for sudden cardiac death

**DOI:** 10.1093/europace/euy091

**Published:** 2018-06-14

**Authors:** Valentina Kutyifa, Katherine Vermilye, Usama A Daimee, Scott McNitt, Helmut Klein, Arthur J Moss

**Affiliations:** Cardiology Division, Heart Research Follow-up Program, University of Rochester Medical Center, 265 Crittenden Blvd., Box 653, Rochester, NY, USA

**Keywords:** Wearable cardioverter-defibrillator, Extended use, Sudden cardiac death, Outcomes

## Abstract

**Aims:**

Data on outcomes in patients using the wearable cardioverter-defibrillator (WCD) > 90 days are limited. We aimed to analyse the clinical course of patients with WCD use ≤90 days vs. WCD use >90 days.

**Methods and results:**

We assessed arrhythmia events during WCD use, and ejection fraction (EF) improvement/implantable cardioverter-defibrillator (ICD) implantation at the end of WCD use in patients with WCD use ≤90 days vs. WCD use >90 days enrolled in the WEARIT-II registry, further assessed by disease aetiology (ischaemic vs. non-ischaemic vs. congenital/inherited heart disease). There were 981 (49%) patients with WCD use >90 days, and 1019 patients with WCD use ≤90 days (median 120 vs. 55 days). There was a lower incidence of sustained ventricular tachycardia/ventricular fibrillation (VT/VF) events (11 vs. 50 events per 100 patient-years, *P* < 0.001), WCD treated VT/VF events (1 vs. 8 events per 100 patient-years, *P* < 0.001), and non-sustained VT events (21 vs. 51 events per 100 patient-years, *P* = 0.008) with WCD use >90 vs. WCD use ≤90 days. Non-ischaemic cardiomyopathy patients presented with similar rates of sustained VT/VF events during WCD use >90 vs. ≤90 days (13.4 vs. 13.7 events per 100 patient-years, *P* = 0.314), while most of these events terminated spontaneously. One-third of the patients with extended WCD use further improved their EF and they were not implanted with an ICD, with similar rates among ischaemic and non-ischaemic patients.

**Conclusions:**

In WEARIT-II, patients with extended WCD use >90 days remain at risk for ventricular arrhythmia events. One-third of the patients with WCD use >90 days further improved their EF, avoiding the need to consider ICD implantation.

**What’s new?**

- We have described wearable cardioverter-defibrillator (WCD) use >90 days in a large cohort of patients prescribed the WCD in a real-life registry.
- We showed a lower incidence of sustained ventricular tachycardia/ventricular fibrillation (VT/VF) events, WCD treated VT/VF events and non-sustained VT events with WCD use >90 vs. WCD use ≤90 days.
- Non-ischaemic patients had similar rates of sustained VT/VF events during WCD use >90 vs. ≤90 days, while most of these events terminated spontaneously.
- One-third of the patients with extended WCD use improved their ejection fraction (EF), and they were not implanted with an implantable cardioverter-defibrillator.
- Congenital/inherited patients were less likely to improve their EF even with prolonged use of WCD > 90 days.

## Introduction

Wearable cardioverter-defibrillators (WCDs) are currently used in patients at risk for sudden cardiac death (SCD) who are temporarily unable to receive an implantable cardioverter-defibrillator (ICD), or when the risk for SCD may improve over time.^1–3^ Wearable cardioverter-defibrillators are safe and effective in terminating VT/VF events and provide protection against SCD while awaiting implantation of an ICD, or during a time of risk stratification.^4^^,^^5^ Current recommendations on the use of the WCD suggest 40 or 90 days as the mandatory ICD waiting periods following a myocardial infarction (MI) or percutaneous coronary intervention (PCI)/coronary artery bypass graft surgery (CABG), respectively, and 90-day wait period for newly diagnosed non-ischaemic cardiomyopathy patients.

Little is known about WCD use >90 days and its outcomes. There might be a conceivable benefit from an extended use of the WCD in certain populations, especially in patients with non-ischaemic cardiomyopathy (NICM), who often improve their ejection fraction (EF) during a prolonged period of time.^6^ In the WEARIT-II Registry, the first prospective large registry of the WCD, there were a large number of patients with WCD use >90 days, providing us with a unique opportunity for analysis.^7^

Therefore, the aim of the present sub-study of the WEARIT-II Prospective Registry on the WCD was (i) to assess the characteristics of prolonged WCD use >90 days, (ii) to analyse ventricular arrhythmia events during WCD use, and to assess end of WCD use outcomes of either improvement in EF or ICD implantation in patients with WCD use ≤90 days vs. >90 days, and (iii) to provide disease aetiology-specific information on WCD use ≤90 days vs. >90 days and its outcomes.

## Methods

### Patient population

All patients who were medically prescribed a WCD (LifeVest system, ZOLL, Pittsburgh, PA, USA) received an offer to participate in the Registry in the form of a recruitment letter included with the WCD, as reported previously.^7^ Current indication for the use of the WCD has been outlined previously. In short, patients with low EF at risk for SCD after MI, following coronary revascularization (PCI/CABG), with new-onset dilated NICM, or with inherited or congenital heart disease (C/I) were prescribed the WCD for a temporary time period, while they were not candidates for an ICD. High risk for SCD included adult patients with significantly reduced left ventricular function 40 days following a MI, 90 days following a PCI or coronary artery bypass graft surgery (CABG), typically 90 days or longer for newly diagnosed non-ischaemic cardiomyopathy starting guideline directed medical therapy, as well as congenital/inherited heart disease patients at high risk for SCD (e.g. SCD in family history) during a time period of risk stratification (e.g. EPS testing, genetic testing). Recommendations for WCD use have been summarized by the AHA consensus document^2^ and outlined by the 2015 ESC guidelines for the management of patients with ventricular arrhythmias and the prevention of SCD.^8^ Patients who agreed to participate were entered into the Registry after written informed consent. Patients in the Registry received standard medical treatment according to guidelines, and the Registry physicians were not involved in any medical care of the subjects. The University of Rochester, Rochester, NY, USA was the Coordination and Data Center for the WEARIT-II Registry, responsible for the overall study and data management of the Registry. The study protocol was approved by the Research Subjects Review Board at the University of Rochester, Rochester, NY, USA.

### Data collection and follow-up

Baseline medical history and comorbidities were collected through patient questionnaires at enrolment in the Registry. We also collected information on the indication for WCD use from the medical order forms. Further data were collected from the WCD devices, including arrhythmia events, WCD shocks delivered, and hours per day of WCD use. Follow-up questionnaires were sent to patients and physicians at 3 and 12 months post-enrolment. At the end of WCD use, patients were assessed for reasons for discontinuation, such as ICD implantation or EF improvement.

### Definitions and endpoints

Patients were divided into two categories based on the duration of WCD use: WCD use ≤90 days, and >90 days, using the actual wear time recorded in the Registry. This study focused on events during WCD use and end of use reasons at the end of WCD use.

Patients were assigned to the following subgroups based on disease aetiology: (i) patients who had ischaemic cardiomyopathy (ICM) with previous MI or known coronary artery disease with a high risk for SCD, (ii) patients who had a newly diagnosed non-ischaemic cardiomyopathy (NICM) with no known coronary artery disease, and (iii) patients who had congenital/inherited (C/I) heart disease with low EF and high risk for SCD.

The endpoints of the current study were ventricular arrhythmia events, such as sustained ventricular tachycardia (VT) or ventricular fibrillation (VF) with or without WCD shock, and non-sustained VT events. At the end of the WCD use, we analysed ICD implantation rate vs. EF improvement by WCD use duration in the total population, as well as by disease aetiology. A number of patients had reasons ‘other’ than EF improvement or ICD implantation for WCD use termination. Other included cases when the WCD was returned due to condition deterioration, patient decision, insurance denial, death, non-compliance, skin condition, non-response, or WCD return without a specified reason.

### Ventricular arrhythmia events

A ventricular arrhythmia episode that was separated by 5 min from the previous event was considered a separate episode. Each individual ventricular arrhythmia episode was reviewed and adjudicated in the registry and classified into three major categories: (i) sustained VT (lasting 30 s or longer) or VF with WCD shock therapy, (ii) sustained VT with no WCD shock delivered due to the use of the response buttons in the case of haemodynamically stable/self-terminating VTs, and (iii) non-sustained VT of <30 s in duration. Bradyarrhythmia events and inappropriate WCD shocks were rare, and they were not considered as endpoints for this analysis.

### Statistical analysis

Baseline clinical characteristics were compared between patients with WCD use ≤90 days, and >90 days. Categorical variables were analysed using the χ^2^ or Fisher’s exact test and were expressed as frequencies and percentages. Continuous variables were analysed using the non-parametric Rank-Sum Test, and were expressed as median and interquartile range, as appropriate.

Ventricular arrhythmia events were reported for patients with WCD use ≤90 days, and >90 days by displaying the number and percentage of patients with each event. When comparing the incidence of ventricular arrhythmias, we calculated the number of events per 100 patient-years. Comparison of event rates was performed using negative binomial regression tests. Outcomes at the end of WCD use such as ICD implantation vs. EF improvement were expressed by WCD use ≤90 days vs. >90 days as number and percentages and they were compared using the χ^2^ test.

Within ischaemic cardiomyopathy, non-ischaemic cardiomyopathy, and congenital/inherited heart disease patients, specific analyses were performed to assess the rate of arrhythmia events, as well as ICD implantation and EF improvement in patients with WCD use ≤90 days and >90 days using the tests described above.

Analyses were performed using SAS 9.4 statistical software (Cary, NC, USA). All statistical tests performed were two-sided. A *P*-value of <0.05 was considered statistically significant.

## Results

**Figure 1:**
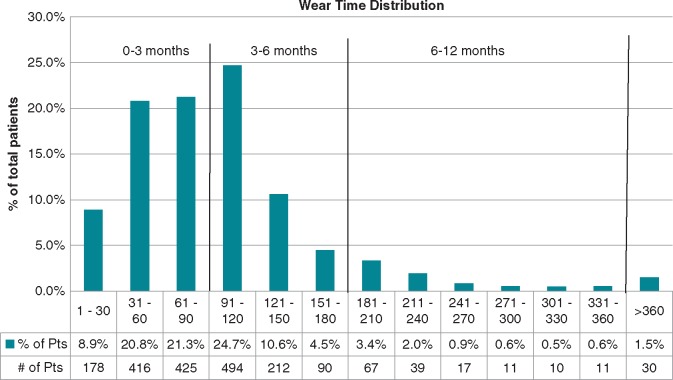
Distribution of WCD use days in WEARIT-II. WCD, wearable cardioverter-defibrillator.

Of the 2000 patients included in the WEARIT-II registry, 981 patients (49%) used the WCD for greater than 90 days. Of patients with WCD use >90 days, 494 patients (50%) had WCD use between 91–120 days, and 487 patients (50%) had WCD use >20 days. Only a minority of patients used the WCD for over a year (*n* = 30, 1.5%) (*Figure 1*).

### Baseline clinical characteristics

In the extended WCD use >90 days group, patients more often had non-ischaemic cardiomyopathy compared to patients with WCD use ≤90 days (50% vs. 43%, *P* < 0.001). Furthermore, extended WCD use patients more often reported heart failure symptoms at baseline, but they less often had atrial fibrillation, hyperlipidaemia, prior MI, or CABG. However, patients in the WCD ≤ 90 days group were older, with higher EF. Patients with prolonged WCD use were more often non-white, and interestingly, ACE/ARB and aldosterone antagonist use was more frequent in the prolonged WCD use group (*Table 1*).

**Table 1:** Baseline clinical characteristics by WCD use 90 days

Demographics	WCD ≤ 90 days	WCD > 90 days	P-value
Number of patients	1019	981	
Aetiology: ischaemic (ICM)	434 (43)	371 (38)	**0.03**
Non-ischaemic (NICM)	434 (43)	493 (50)	**<0.001**
Congenital/inherited (C/I)	151 (15)	117 (12)	0.057
Age	63 (16)	61 (17)	**0.011**
Female gender	302 (30)	296 (30)	0.79
White race	901 (88)	790 (81)	**<0.001**
Ejection fraction	30 (15)	25 (15)	**0.015**
Hispanic	31 (3)	41 (4)	0.39
Daily use (h)	22.2 (2.6)	22.5 (3.3)	**0.001**
Heart failure at baseline	481 (47)	559 (57)	**<0.001**
Atrial fibrillation	316 (31)	241 (25)	**0.001**
Hypertension	635 (62)	573 (58)	0.07
Hyperlipidaemia	539 (53)	456 (46)	**0.004**
Diabetes	296 (29)	255 (26)	0.12
Renal disease	79 (8)	83 (8)	0.56
Myocardial infarction	479 (47)	406 (41)	**0.011**
Percutaneous coronary angioplasty	335 (33)	275 (28)	**0.019**
CABG	183 (18)	139 (14)	**0.021**
Cardiomyopathy	434 (43)	465 (47)	**0.031**
Aldosterone antagonist	243 (24)	318 (32)	**<0.001**
ACE-I/ARB	714 (70)	768 (78)	**<0.001**
Beta-blockers	871 (85)	859 (88)	0.17
Amiodarone	142 (14)	117 (12)	0.18

Data are expressed as number and percentages and median and interquartile ranges. *P*-values < 0.05 are highlighted in bold.

ACE-I, angiotensin converting enzyme inhibitor; ARB, angiotensin receptor blocker; CABG, coronary artery bypass grafting; C/I, congenital or inherited condition; ICM, ischaemic cardiomyopathy; NICM, non-ischaemic cardiomyopathy; WCD, wearable cardioverter-defibrillator.

### Ventricular arrhythmia events by wearable cardioverter-defibrillator use duration

In patients with WCD use >90 days, there was a lower incidence of any sustained VT/VF (13 vs. 28 patients, 11 vs. 50 events per 100 patient-years, *P* < 0.001), sustained VT events without treatment (10 vs. 12 patients, 10 vs. 32 events per 100 patient-years, *P* = 0.008), and sustained VT/VF treated with WCD shock (3 vs. 19 patients, 1 vs. 18 events per 100 patient-years, *P* < 0.001) as compared to WCD use ≤90 days. Similarly, the incidence of non-sustained VT events was lower with WCD use >90 days (51 vs. 21 events per 100 patient-years, *P* = 0.030) (*Table 2*). When we assessed the timing of the events in patients with WCD use >90 days, there were six patients with any sustained VT/VF before 90 days of use, and seven patients with any sustained VT/VF after 90 days. Five of the 10 patients had sustained VT events without treatment before 90 days, and the other five had them after 90 days. However, two of the three treated VT/VF events occurred past 90 days of use.

**Table 2:** Arrhythmia events during WCD use by ≤90 days vs. >90 days

Event	Patients, *n* (%)	Events (mean events/ patient)	Event rate per 100 patient-years	*P*-value for ≤90 days vs. >90 days
WCD use ≤90 days				
All sustained VT/VF	28 (2.7)	76 (2.7)	50	**<0.001**
Sustained VT no treatment	12 (1.2)	49 (4.1)	32	**0.008**
Sustained VT treated	19 (1.9)	27 (1.4)	18	**<0.001**
NSVT	12 (1.2)	78 (6.5)	51	**0.030**
WCD use >90 days				
All sustained VT/VF	13 (1.3)	44 (3.4)	11	<0.001
Sustained VT no treatment	10 (1.0)	41 (4.1)	10	0.008
Sustained VT treated	3 (0.3)	3 (1)	1	<0.001
NSVT	16 (1.6)	86 (5.4)	21	0.030

*P*-values < 0.05 are highlighted in bold.

NSVT, non-sustained ventricular tachycardia; VT/VF, ventricular tachycardia/ventricular fibrillation; WCD, wearable cardioverter-defibrillator.

### Ventricular arrhythmia events by wearable cardioverter-defibrillator use duration and by disease aetiology

Among patients with ischaemic cardiomyopathy, those with WCD use >90 days had a lower rate of sustained VT/VF (7 vs. 17 patients, 10.6 vs. 56.8 events per 100 patient-years, *P* = 0.001), and a lower rate of WCD treated VT/VF (3 vs. 6 patients, 9.3 vs. 35.3 events per 100 patient-years, *P* < 0.001) as compared to WCD use ≤90 days. In patients with non-ischaemic cardiomyopathy, there was a similar rate of sustained VT/VF events in both the WCD use >90 days and WCD use ≤90 days groups (5 vs. 5 patients, 13.4 vs. 13.7 events per 100 patient-years, *P* = 0.314); however, most of these events were self-terminating without WCD shocks. Congenital/inherited heart disease patients also had less sustained VT/VF events during WCD use >90 days, and there were no WCD shocks in this group (*Tables 3* and *4*).

**Table 3:** Ventricular arrhythmia events during WCD use ≤90 days by aetiology

	Patients, *n* (%)	Events (mean events/ patient)	Event rate per 100 patient-years	*P*-value for ≤90 days vs. >90 days
Ischaemic cardiomyopathy				
All sustained VT/VF	17 (4.6)	37 (2.2)	56.8	**0.001**
Sustained VT no treatment	6 (1.6)	23 (3.8)	35.3	0.074
Sustained VT treated	13 (3.5)	14 (1.1)	21.5	**<0.001**
NSVT	3 (0.8)	39 (13)	59.8	0.063
Non-ischaemic cardiomyopathy				
All sustained VT/VF	5 (1.2)	9 (1.8)	13.7	0.314
Sustained VT no treatment	2 (0.5)	6 (3.0)	9.1	0.543
Sustained VT treated	3 (0.7)	3 (1.0)	4.6	0.055
NSVT	5 (1.2)	22 (4.4)	33.5	0.541
Congenital/inherited heart disease patients				
All sustained VT/VF	6 (4)	30 (5.0)	135.9	**0.003**
Sustained VT no treatment	4 (2.7)	20 (5.0)	90.6	**0.013**
Sustained VT treated	3 (2)	10 (3.3)	45.3	n.a.
NSVT	4 (2.6)	17 (4.3)	77.0	**0.043**

*P*-values < 0.05 are highlighted in bold.

NSVT, non-sustained ventricular tachycardia; VT/VF, ventricular tachycardia/ventricular fibrillation; WCD, wearable cardioverter-defibrillator.

**Table 4:** Ventricular arrhythmia events during WCD use >90 days by aetiology

	Patients, *n* (%)	Events (mean events/ patient)	Event rate per 100 patient-years	*P*-value for ≤90 days vs. >90 days
Ischaemic cardiomyopathy				
All sustained VT/VF	7 (1.9)	16 (2.3)	10.6	**0.001**
Sustained VT no treatment	5 (1.3)	2 (0.4)	1.3	0.074
Sustained VT treated	2 (0.5)	14 (7)	9.3	**<0.001**
NSVT	5 (1.3)	12 (2.4)	7.9	0.063
Non-ischaemic cardiomyopathy				
All sustained VT/VF	5 (1)	27 (5.4)	13.4	0.314
Sustained VT no treatment	4 (0.8)	26 (6.5)	12.9	0.543
Sustained VT treated	1 (0.2)	1 (1.0)	0.5	0.055
NSVT	7 (1.4)	69 (9.9)	34.3	0.541
Congenital/inherited heart disease patients				
All sustained VT/VF	1 (0.9)	1 (1)	2.0	**0.003**
Sustained VT no treatment	1 (0.9)	1 (1)	2.0	**0.013**
Sustained VT treated	0 (0)	0 (0.0)	0.0	n.a.
NSVT	4 (3.4)	5 (1.3)	10.1	**0.043**

*P*-values < 0.05 are highlighted in bold.

NSVT, non-sustained ventricular tachycardia; VT/VF, ventricular tachycardia/ventricular fibrillation; WCD, wearable cardioverter-defibrillator.

### End-of-use outcomes by wearable cardioverter-defibrillator use duration

**Figure 2:**
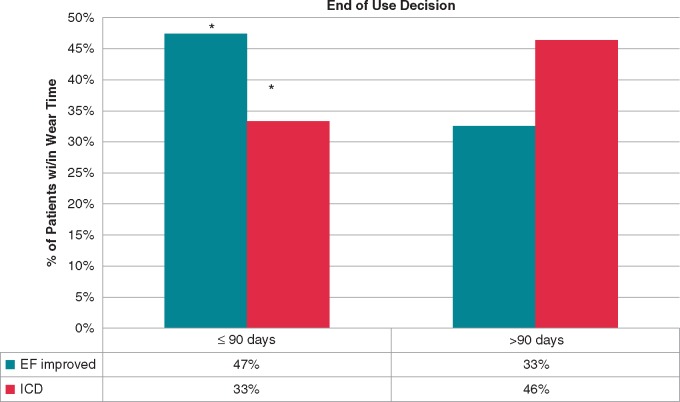
Clinical outcomes at the end of WCD use by duration of WCD use. *P < 0.001 for end of use category for patients with ≤90 days of total wear vs. >90 days of total wear. EF, ejection fraction; ICD, received implantable cardioverter-defibrillator; WCD, wearable cardioverter-defibrillator.

**Figure 3:**
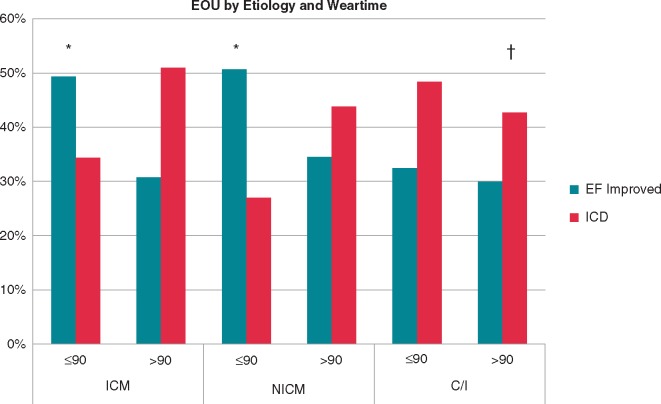
Clinical outcomes at the end of WCD use by the duration of WCD use and by disease aetiology. *P-value <0.001 for ≤90 vs. >90 total days wear time. ^†^*P*-value <0.001 in the ≤90 days wear time group for EOU by disease aetiology. C/I, congenital or inherited heart disease; EF, ejection fraction; EOU, end of use; ICD, implantable cardioverter-defibrillator; ICM, ischaemic cardiomyopathy; NICM, non-ischaemic cardiomyopathy; WCD, wearable cardioverter-defibrillator.

Among patients with WCD use ≤90 days, 33% were implanted with an ICD at the end of WCD use, while 47% of them had improvement in EF and were not implanted with an ICD. An additional 33% of the patients with prolonged WCD use >90 days improved their EF avoiding the need to consider an ICD implantation (*Figure 2*). This additional observed improvement in EF beyond 90 days was similar for ischaemic (31%), non-ischaemic cardiomyopathy (34%), and congenital/inherited heart disease patients (30%) (*Figure 3*).

## Discussion

Our study provides several novel findings: (i) we characterized a large cohort of almost 1000 patients with WCD use >90 days in a contemporary setting, (ii) demonstrated that patients with extended WCD use >90 days remain at risk for ventricular arrhythmia events, (iii) reported that an additional one-third of the patients with WCD use >90 days to improved their EF, and they were not implanted with an ICD, (iv) EF improvement was similar in patients with ischaemic and non-ischaemic cardiomyopathy, and (v) congenital/inherited heart disease patients less often improved their EF and were more often implanted with an ICD. These new findings altogether suggest that WCD use >90 days could potentially improve risk stratification for an ICD in ischaemic and non-ischaemic cardiomyopathy patients.

Previous studies reported on the duration of WCD use and a number of these studies included patients who used the WCD for >90 days. Chung *et al.*^5^ showed an average 52.6 days of WCD use in the national aggregate experience of 3569 patients prescribed the WCD. In their study, only a small proportion of patients used the WCD for >90 days, and they did not specifically report outcomes of the cohort with prolonged WCD use. In another study focusing on post-MI patients, Epstein *et al.*^9^ showed an average of 69 days WCD use in 8453 patients, including time to first appropriate WCD shock as part of their analysis. They found that 96% of first appropriate shocks occurred within the first 90 days of use. However, this study focused only on patients prescribed the WCD within 3 months of an MI, they did not enrol patients with non-ischaemic cardiomyopathy.

So far, very few studies investigated WCD use and its outcomes past 90 days,^5^^,^^6^ and some of these studies reported on longer use in specific subpopulations of cardiomyopathy, or in a very small number of patients.^10^^,^^11^ The PROLONG study by Duncker *et al.*^12^ evaluated prolonged WCD use in 74 of 156 patients, 12 of these patients had WCD shocks, two of them past 90 days of use. Similar findings were revealed in a subsequent report from the same group, focusing on non-ischaemic cardiomyopathy patients only.^13^ In the WEARIT-II Registry, we had almost 1000 subjects, and half of our patients were using the WCD for longer than 90 days. And while the majority of the patients in the WCD use >90 days subgroup had less than 6 months of WCD use duration, our study is the largest to date to report on patients with prolonged WCD use.

Why is our study clinically relevant? We have shown that extended WCD use was associated with a decision of not to implant an ICD in one-third of these patients with ischaemic or non-ischaemic cardiomyopathy. It is conceivable that patients with extended WCD use had a longer time for therapy optimization and left ventricular function recovery as compared to those with WCD use ≤90 days. And although ICDs are lifesaving devices for patients with permanent high risk for SCD, they are associated with an increased risk for lead failure, infection, and inappropriate shocks that could impair quality of life and outcomes, and increase health care costs.^14–18^ Therefore, improved risk stratification with extended WCD use in high-risk cardiomyopathy patients could have cost benefits. Although we do not have detailed data on costs in the WEARIT-II registry, the WCD has been previously shown to be cost-effective in post-MI patients,^19^ and following an ICD infection.^2^

In this sub-study from WEARIT-II, we have shown that at-risk cardiomyopathy patients remain at risk for ventricular arrhythmias during extended WCD use. While the risk of ventricular arrhythmias was shown to be lower for the WCD use >90 days group, it is not a negligible risk. Non-ischaemic cardiomyopathy patients presented with similar rates of sustained VT/VF events with WCD use ≤ 90 days and > 90 days although most of these events were self-terminated. Nevertheless, even self-terminating events are useful in facilitating the decision for an ICD implantation as we have previously shown in the primary report of WEARIT-II.^7^ A total of 65% of patients with sustained VT events not requiring WCD shock were implanted with an ICD at the end of WCD use. This highlights the potential role of the WCD in not only protecting patients from SCD but also monitoring sustained VT events, and such events are useful for risk stratification.

Prolonged WCD use was associated with similar improvement in EF in both ischaemic and non-ischaemic cardiomyopathy patients suggesting that the myocardial substrate is yet to change with longer time for therapy optimization. Patients with congenital/inherited heart disease however had similar rates of EF improvement and ICD implantation with both ≤90 days and >90 days. Whether left ventricular function would further improve with longer WCD use over 6 months or over a year is not fully understood. A prior small study on WCD use >1 year among 220 patients reported 16% of these patients to have EF recovery, and 4.1% to receive an appropriate WCD shock.^20^

Our data on extended WCD use and its outcomes can aid physicians with patient management. Clinicians using the WCD in ischaemic and non-ischaemic cardiomyopathy patients could consider extended use of the WCD in patients in whom there were no ventricular arrhythmia events or EF recovery, especially when therapy optimization is still underway. An extended WCD use strategy for ICD risk stratification however needs to be prospectively assessed. A prospective clinical study assessing extended WCD use outcomes including ventricular arrhythmia rates, ICD implantation, and EF improvement, would provide further insights into additional risk stratification for an ICD. Such a study might be warranted in both patients with ischaemic and non-ischaemic cardiomyopathy, since both of these cohorts showed further EF improvement in our study.

### Limitations

Our study nevertheless has certain limitations. The WEARIT-II registry is a voluntary, observational registry, patients were not randomized, and there was no control group. The registry mainly relied on direct patient reported data collected through surveys that have inherent limitations. However, days of wear and events while using the WCD were recorded from data collected from the WCD devices and were consistently reported for all patients in the registry. In patients with extended WCD use, we did not have detailed information on physician decision for longer use, and such decisions could have been influenced by other factors, such as difficulty in scheduling ICD implantation, changes in patient condition, or patient preferences. Collecting such information might be useful in future studies. As physician decision regarding prolongation of WCD use was not standardized, individual clinical scenarios might have played a role that could affect outcomes, introducing bias for comparison. We did not have detailed information on the specific aetiology of the non-ischaemic patient group. Finally, fewer than 10% of patients in the WEARIT-II registry wore the WCD for >6 months, making it difficult to draw conclusions about extended WCD use >6 months.

## Conclusions

In the WEARIT-II Registry, almost half of the patients had an extended WCD use >90 days. Patients chosen by their physicians for an extended WCD use >90 days remained at risk for ventricular arrhythmias >90 days. In non-ischaemic cardiomyopathy patients, the rate of sustained VT events was similar with standard or extended WCD use, while most of these events were self-terminating. Approximately one-third of ischaemic and non-ischaemic cardiomyopathy patients with WCD use >90 days had an EF improvement, avoiding the need to consider an ICD implantation. Further research is warranted to investigate the utility of prolonged WCD use past 90 days of use with detailed assessment of extended use decisions in ischaemic and non-ischaemic patients.

## References

[1] Chung MK. The role of the wearable cardioverter defibrillator in clinical practice. Cardiol Clin 2014;32:253–70. 24793801 10.1016/j.ccl.2013.11.002

[2] Piccini JP Sr, Allen LA, Kudenchuk PJ, Page RL, Patel MR, Turakhia MP. Wearable cardioverter-defibrillator therapy for the prevention of sudden cardiac death: a science advisory from the American Heart Association. Circulation 2016;133:1715–27. 27022063 10.1161/CIR.0000000000000394

[3] Reek S, Burri H, Roberts PR, Perings C, Epstein AE, Klein HU et al The wearable cardioverter-defibrillator: current technology and evolving indications. Europace 2017;19:335–45. 27702851 10.1093/europace/euw180

[4] Feldman AM, Klein H, Tchou P, Murali S, Hall WJ, Mancini D et al Use of a wearable defibrillator in terminating tachyarrhythmias in patients at high risk for sudden death: results of the WEARIT/BIROAD. Pacing Clin Electrophysiol 2004;27:4–9. 14720148 10.1111/j.1540-8159.2004.00378.x

[5] Chung MK, Szymkiewicz SJ, Shao M, Zishiri E, Niebauer MJ, Lindsay BD et al Aggregate national experience with the wearable cardioverter-defibrillator: event rates, compliance, and survival. J Am Coll Cardiol 2010;56:194–203. 20620738 10.1016/j.jacc.2010.04.016 PMC2962668

[6] Salehi N, Nasiri M, Bianco NR, Opreanu M, Singh V, Satija V et al The wearable cardioverter defibrillator in nonischemic cardiomyopathy: a US National Database Analysis. Can J Cardiol 2016;32:1247.e1–1247.e6. 10.1016/j.cjca.2015.12.035 26975224

[7] Kutyifa V, Moss AJ, Klein H, Biton Y, McNitt S, MacKecknie B et al Use of the wearable cardioverter defibrillator in high-risk cardiac patients: data from the Prospective Registry of Patients Using the Wearable Cardioverter Defibrillator (WEARIT-II Registry). Circulation 2015;132:1613–9. 26316618 10.1161/CIRCULATIONAHA.115.015677

[8] Priori SG, Blomstrom-Lundqvist C, Mazzanti A, Blom N, Borggrefe M, Camm J et al 2015 ESC guidelines for the management of patients with ventricular arrhythmias and the prevention of sudden cardiac death: the Task Force for the Management of Patients with Ventricular Arrhythmias and the Prevention of Sudden Cardiac Death of the European Society of Cardiology (ESC) Endorsed by: Association for European Paediatric and Congenital Cardiology (AEPC). Europace 2015;17:1601–87. 26318695 10.1093/europace/euv319

[9] Epstein AE, Abraham WT, Bianco N, Kern KB, Mirro M, Rao SV et al Wearable cardioverter-defibrillator use in patients perceived to be at high risk early post myocardial infarction. J Am Coll Cardiol 2013;62:2000–7. 23916930 10.1016/j.jacc.2013.05.086

[10] Duncker D, Haghikia A, Konig T, Hohmann S, Gutleben KJ, Westenfeld R et al Risk for ventricular fibrillation in peripartum cardiomyopathy with severely reduced left ventricular function-value of the wearable cardioverter/defibrillator. Eur J Heart Fail 2014;16:1331–6. 25371320 10.1002/ejhf.188

[11] Deeprasertkul P, Opreanu M, Bianco N, Thakur R. National experience with wearable cardioverter-defibrillator use in takotsubo cardiomyopathy. J Am Coll Cardiol (JACC) 2013;61:E361.

[12] Duncker D, Konig T, Hohmann S, Bauersachs J, Veltmann C. Avoiding untimely implantable cardioverter/defibrillator implantation by intensified heart failure therapy optimization supported by the wearable cardioverter/defibrillator—the PROLONG study. J Am Heart Assoc 2017;6:e004512. 28096098 10.1161/JAHA.116.004512 PMC5523634

[13] Duncker D, Konig T, Hohmann S, Bauersachs J, Veltmann C. Ventricular arrhythmias in patients with newly diagnosed nonischemic cardiomyopathy: insights from the PROLONG study. Clin Cardiol 2017;40:586–90. 28333373 10.1002/clc.22706 PMC6490321

[14] Groarke JD, Buckley U, Collison D, O’Neill J, Mahon NG, Foley B. Cost implications of defibrillator lead failures. Europace 2012;14:1156–60. 22333240 10.1093/europace/eus006

[15] Greenspon AJ, Patel JD, Lau E, Ochoa JA, Frisch DR, Ho RT et al 16-year trends in the infection burden for pacemakers and implantable cardioverter-defibrillators in the United States: 1993 to 2008. J Am Coll Cardiol 2011;58:1001–6. 21867833 10.1016/j.jacc.2011.04.033

[16] Sohail MR, Henrikson CA, Braid-Forbes M, Forbes KF, Lerner DJ. Mortality and cost associated with cardiovascular implantable electronic device infections. Arch Intern Med 2011;171:1821–8. 21911623 10.1001/archinternmed.2011.441

[17] Reynolds MR, Cohen DJ, Kugelmass AD, Brown PP, Becker ER, Culler SD et al The frequency and incremental cost of major complications among medicare beneficiaries receiving implantable cardioverter-defibrillators. J Am Coll Cardiol 2006;47:2493–7. 16781379 10.1016/j.jacc.2006.02.049 PMC1800827

[18] Poole JE, Johnson GW, Hellkamp AS, Anderson J, Callans DJ, Raitt MH et al Prognostic importance of defibrillator shocks in patients with heart failure. N Engl J Med 2008;359:1009–17. 18768944 10.1056/NEJMoa071098 PMC2922510

[19] Køber L, Thune JJ, Nielsen JC, Haarbo J, Videbæk L, Korup E et al Defibrillator implantation in patients with nonischemic systolic heart failure. N Engl J Med 2016;375:1221–30. 27571011 10.1056/NEJMoa1608029

[20] Lamichhane M, Gardiner JC, Bianco NR, Szymkiewicz SJ, Thakur RK. National experience with long-term use of the wearable cardioverter defibrillator in patients with cardiomyopathy. J Interv Card Electrophysiol 2017;48:11–9. 27752809 10.1007/s10840-016-0194-6

